# Supplementary material for: A Humanized Yeast Phenomic Model of Deoxycytidine Kinase to Predict Genetic Buffering of Nucleoside Analog Cytotoxicity
Source: Genes (Basel). 2019 Sep 30;10(10):770. doi: 10.3390/genes10100770 (PMC6826991; doi:10.3390/genes10100770)

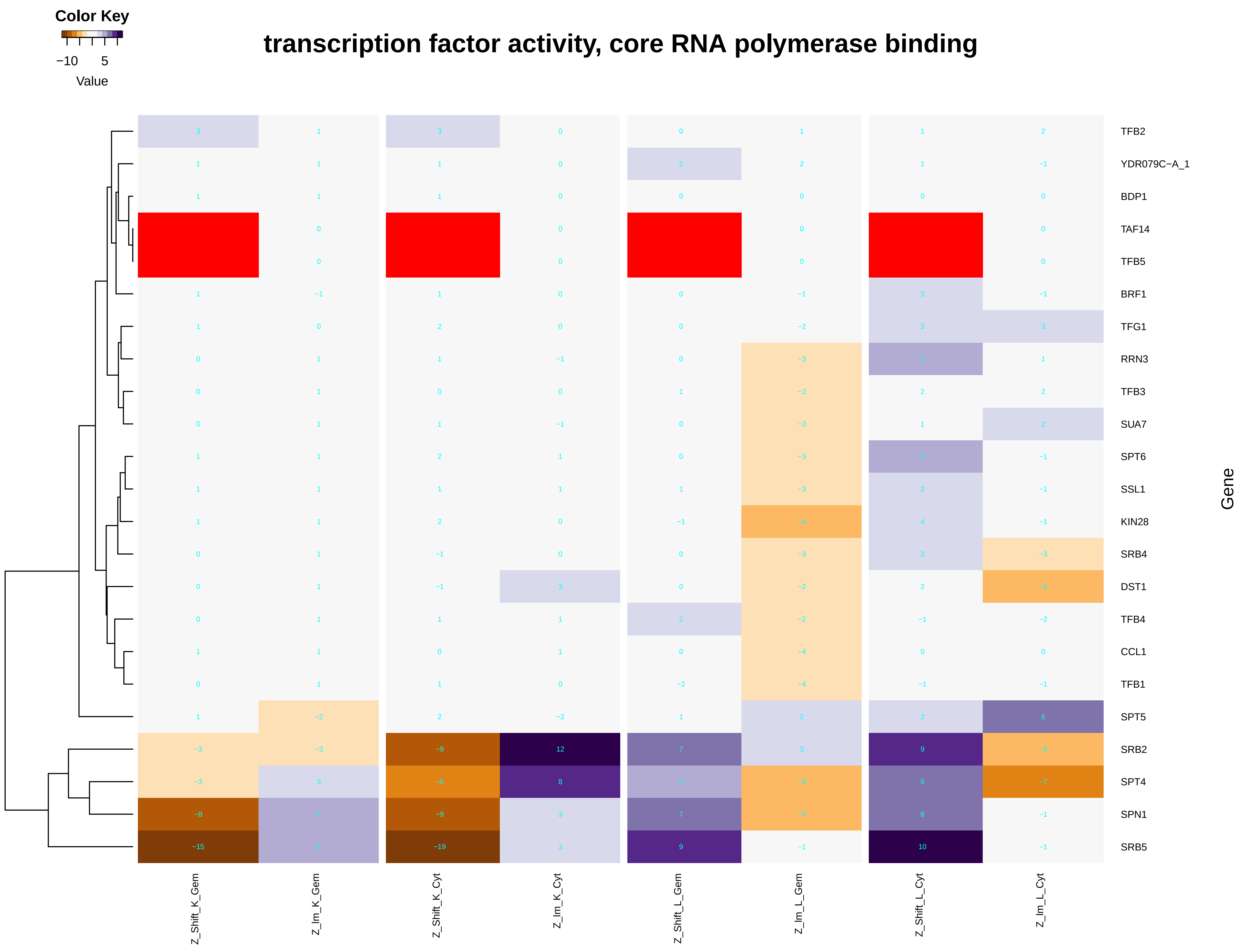

transcription factor activity, core RNA polymerase binding
Color Key
−10 5
Value
Gene
TFB2
YDR079C−A_1
BDP1
TAF14
TFB5
BRF1
TFG1
RRN3
TFB3
SUA7
SPT6
SSL1
KIN28
SRB4
DST1
TFB4
CCL1
TFB1
SPT5
SRB2
SPT4
SPN1
SRB5
Z_Shift_K_Gem
Z_Im_K_Gem
Z_Shift_K_Cyt
Z_Im_K_Cyt
Z_Shift_L_Gem
Z_Im_L_Gem
Z_Shift_L_Cyt
Z_Im_L_Cyt

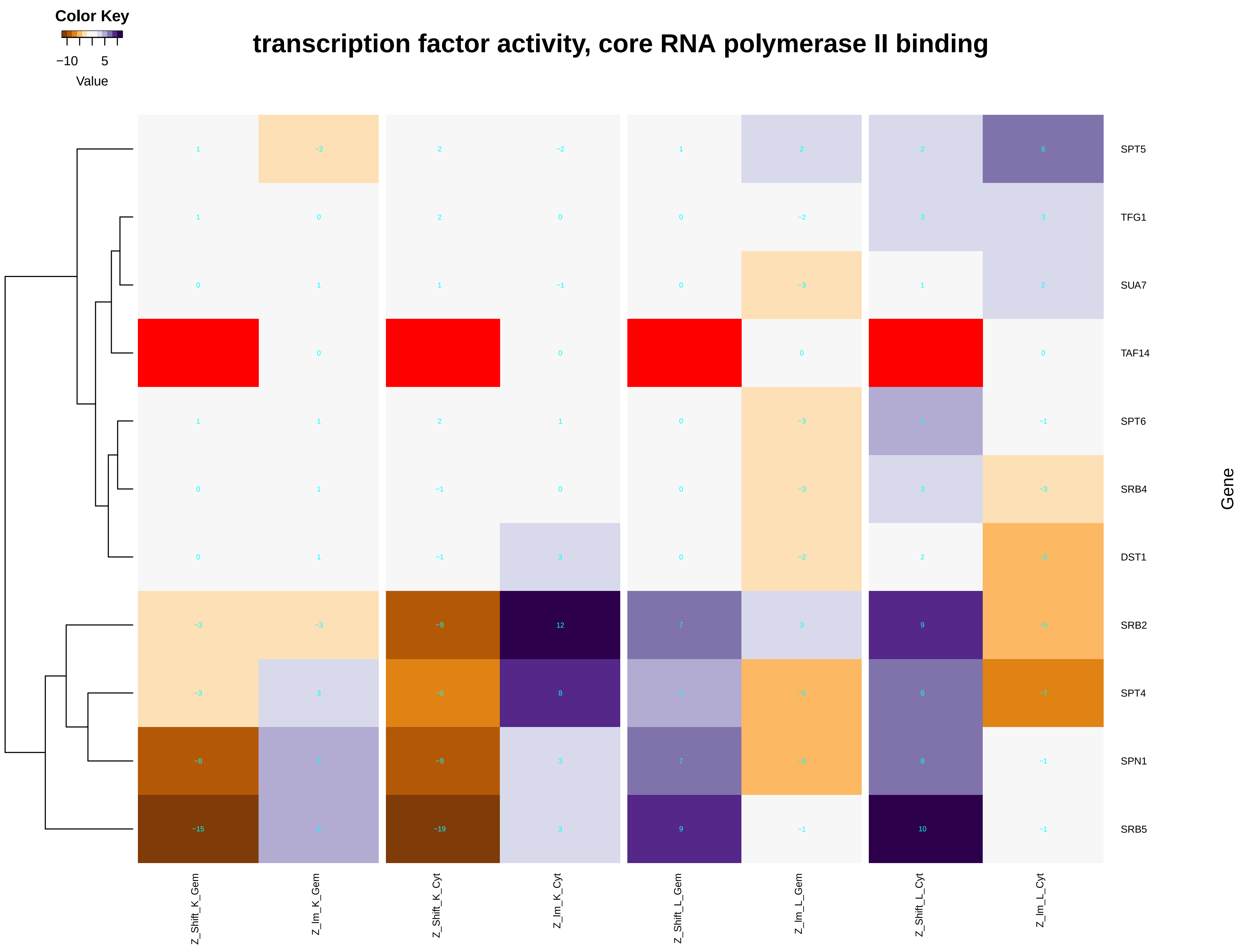
Color Key
−10
5
Value
transcription factor activity, core RNA polymerase II binding
SPT5
TFG1
SUA7
TAF14
SPT6
SRB4
DST1
SRB2
SPT4
SPN1
SRB5
Gene
Z_Shift_K_Gem
Z_Im_K_Gem
Z_Shift_K_Cyt
Z_Im_K_Cyt
Z_Shift_L_Gem
Z_Im_L_Gem
Z_Shift_L_Cyt
Z_Im_L_Cyt

transcription factor activity, core RNA polymerase I binding

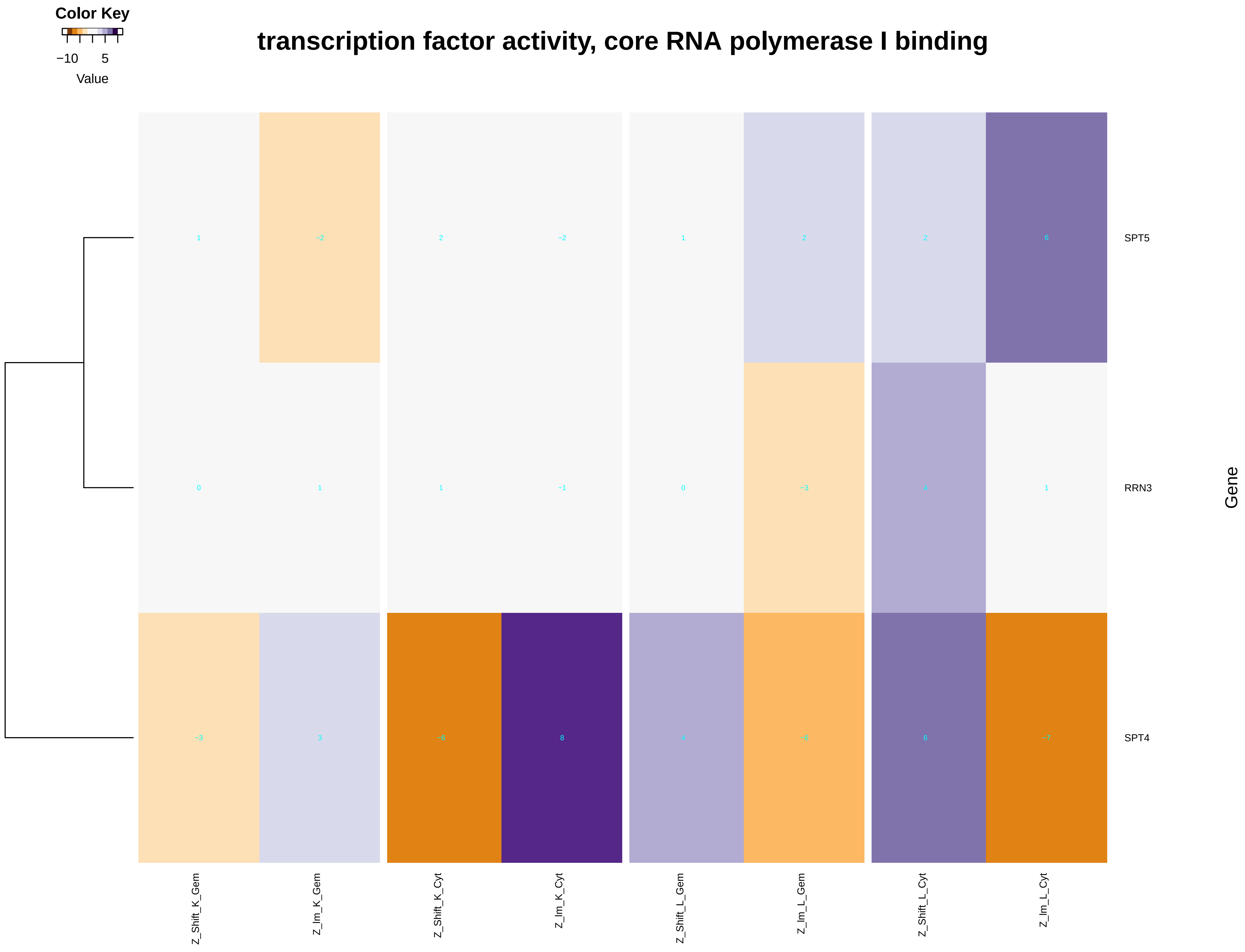

transcription factor activity, core RNA polymerase II recruiting

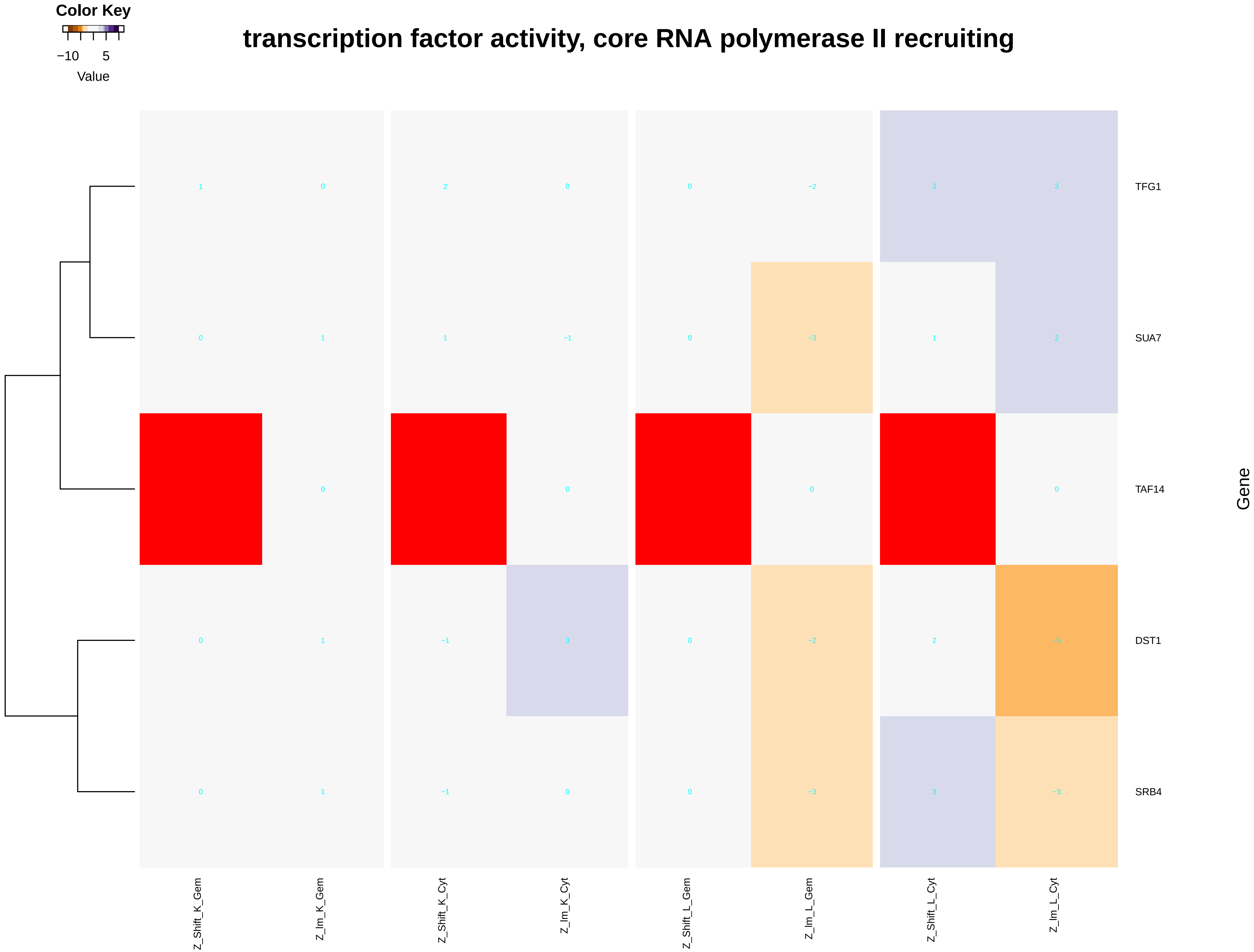

Supplement: Supplementary file 1 [file genes-10-00770-s001.zip › Additional_Files/Additional_File7_GOTermHeatmaps.zip!/Additional_File7_GOTermHeatmaps/transcription_factor_activity,_core_RNA_polymerase_binding.pdf]
